# Prostatic Abscess by Morganella morganii

**DOI:** 10.7759/cureus.86265

**Published:** 2025-06-18

**Authors:** Minoru Sakakiyama, Koji Hayashi, Miki Tada, Maho Hayashi, Yuka Nakaya, Mamiko Sato, Toshiko Iwasaki, Yasutaka Kobayashi, Katsunori Mizuno

**Affiliations:** 1 Department of Internal Medicine, Fukui General Hospital, Fukui, JPN; 2 Department of Rehabilitation Medicine, Fukui General Hospital, Fukui, JPN; 3 Department of Radiology, Fukui General Hospital, Fukui, JPN; 4 Graduate School of Health Science, Fukui Health Science University, Fukui, JPN; 5 Department of Orthopedics, Fukui General Hospital, Fukui, JPN

**Keywords:** abscess, morganella morganii, postoperation, prostate gland, prostatic abscess, urinary catheter

## Abstract

We describe a case of a prostatic abscess (PA) caused by *Morganella morganii* (*M. morganii*). The patient was a 71-year-old immunocompetent man who was hospitalized due to lumbar surgery. A urethral catheter was placed perioperatively. On postoperative day (POD) 6, the patient developed a fever of 38.5°C, elevated inflammatory markers including leukocytosis and C-reactive protein, and pyuria. Blood and urine cultures were positive for *M. morganii*. Contrast-enhanced computed tomography revealed a hypodense area in the prostate, consistent with PA. The patient was treated with levofloxacin, and his symptoms resolved by POD 11. He was discharged on POD 27 with no signs of recurrence.

PA can exhibit diverse, nonspecific symptoms, necessitating clinical suspicion and thorough diagnostics. Identifying* M. morganii *as the causative agent highlights the need to consider atypical pathogens in postoperative or nosocomial cases, especially in patients with urinary catheters. Further research is needed to explore the pathogenic mechanisms and optimal management of PA caused by uncommon organisms like *M. morganii*, emphasizing that early detection and tailored treatment are vital for improving patient outcomes and reducing complications.

## Introduction

A prostatic abscess (PA) is defined as a localized collection of purulent fluid within the prostate gland [1,2]. It is considered a serious infection of the prostate and can be a complication of acute bacterial prostatitis or result from the hematogenous spread of infection [1-3]. PA is uncommon or rare in clinical practice, particularly since the development and widespread use of effective antibiotic therapy [1,4,5]. They are most often encountered in elderly and compromised patients [6]. Historically, PA was diagnosed in only 0.2% of patients with urologic symptoms and in 0.5-2.5% of patients hospitalized for prostatic symptoms [1,6]. While still rare among young, healthy men, the incidence is increasing in patients with chronic medical conditions [1].

*Morganella morganii* (*M. morganii*) is a Gram-negative, rod-shaped bacillus that is facultatively anaerobic [7,8]. It is classified within the *Enterobacteriaceae* family and the *Proteeae* tribe, which includes species such as *Proteus* and *Providencia* [7-9]. This bacterium was first identified in 1906 from a fecal sample taken from a child [7-9]. The genus *Morganella* currently comprises a single species, *M. morganii*, which has two subspecies: *morganii* and *sibonii* [7,8]. Biologically, *M. morganii *is motile and does not ferment lactose while also being capable of producing urease [10]. It is found widely in various environments and is part of the normal gut microbiota in humans, mammals, and reptiles [7-10].

Historically, *M. morganii *was regarded as clinically insignificant and primarily linked to conditions such as summer diarrhea [7,9]. However, it has since been redefined as a rare opportunistic pathogen [7-9]. This bacterium is mainly identified in nosocomial (hospital-acquired) infections, particularly among postoperative patients [7,9]. Nonetheless, infections can also arise in community settings and among healthy individuals, though these occurrences are rarer [8]. Evidence from around the globe indicates its rising significance as a hospital-associated pathogen, with opportunistic infections reported internationally [7,8].

Regarding pathogenesis, it has been reported that *M. morganii *produces various virulence factors essential for bacterial virulence and infection, including urease, IgA protease, hemolysins, type III secretion system (T3SS), lipopolysaccharide (LPS), fimbrial adhesins, and two-component systems (TCSs) [7,9,10]. Additionally, urease production supports bacterial growth and biofilm formation during urinary tract infections [9]. Furthermore, antibiotic resistance in *M. morganii* is a growing concern. *M. morganii* exhibits intrinsic resistance to several antimicrobial agents. It possesses inherent resistance to penicillins (e.g., ampicillin, amoxicillin), most first- and second-generation cephalosporins (e.g., cefazolin, cefuroxime), macrolides, lincosamides, glycopeptides, fosfomycin, fusidic acid, and colistin [7,9,10]. This intrinsic resistance is often mediated by chromosomally encoded AmpC β-lactamases [7]. Moreover, *M. morganii* can acquire resistance genes via plasmids, transposons, and integrons, facilitating horizontal gene transfer to other bacteria [7,9]. Notably, the presence of extended-spectrum beta-lactamase (ESBL) genes, KPC-2, and NDM-1 significantly complicates the treatment of *M. morganii *infections [7,9]. Thus, the increasing incidence and growing rates of antibiotic resistance suggest that it may be on the verge of becoming the next "superbug" [7]. Therefore, clinicians should consider *M. morganii *as a potential causative agent in their patient evaluations [7,8].

*M. morganii *can lead to a wide range of infections [10], and its disease spectrum is diverse and evolving due to changes in virulence and increasing drug resistance [9]. Traditionally associated with postoperative wound and urinary tract infections [8,9], it can also result in invasive infections at various sites across all age groups, including neonates [7-10]. Documented infection sites and conditions include bloodstream infections, skin and soft tissue infections, urinary tract infections, abscesses (such as renal, brain, abdominal, orbital, liver, tubo-ovarian, and putaminal abscesses), meningitis/meningoencephalitis, and osteoarticular infections [7-10]. Notably, reports of PA associated with *M. morganii *are extremely limited. In this report, we present a unique case of PA attributable to *M. morganii*, highlighting the need for increased awareness and further research on this opportunistic pathogen.

## Case presentation

**Figure 1:**
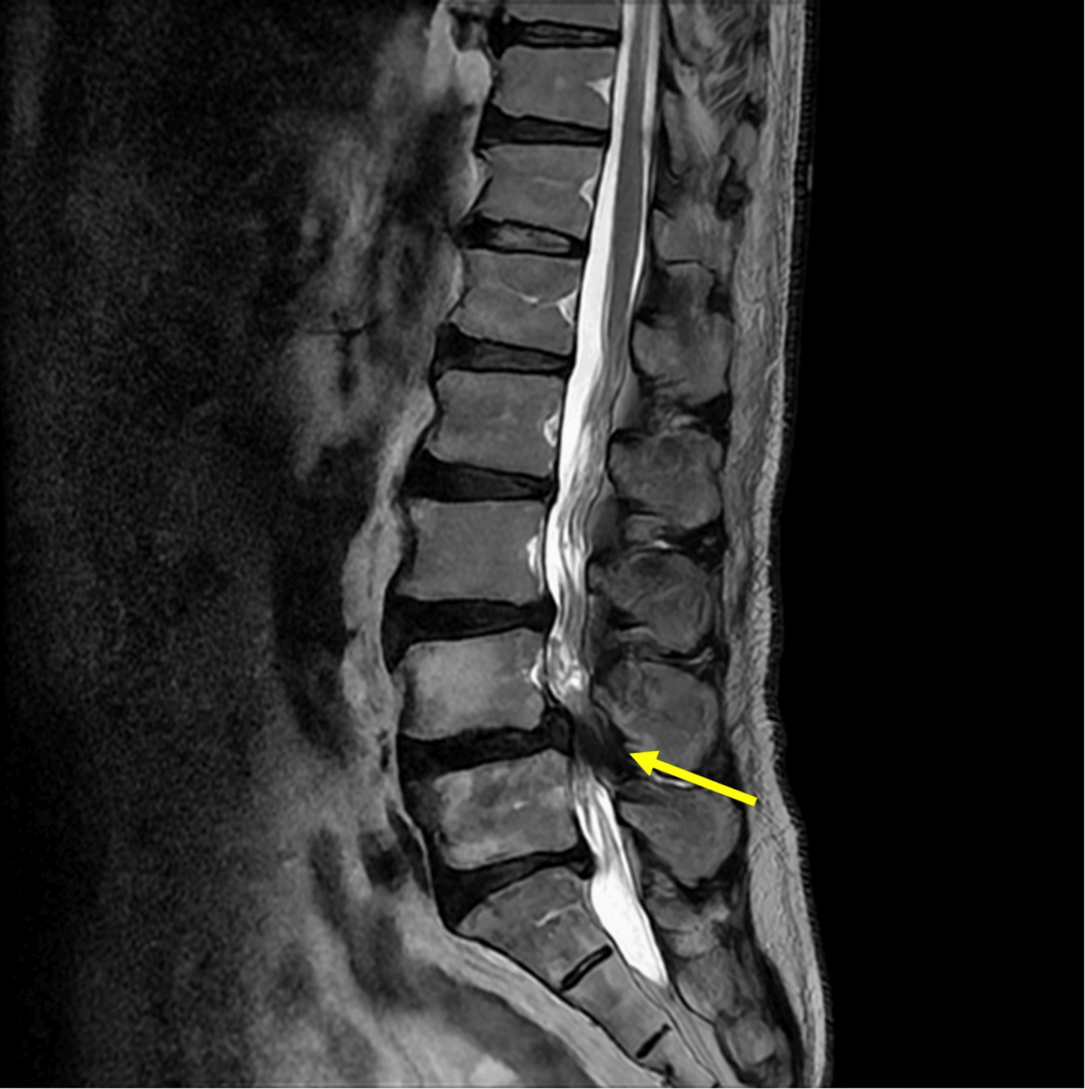
Results of the lumbar MRI. Lumbar MRI showing disc bulging at the L3/4, L4/5, and L5/S1 levels, with prominent stenosis at L4/5 (arrow). MRI: magnetic resonance imaging

A 71-year-old man presented with gait instability and lower extremity pain. He had a history of surgery for subarachnoid hemorrhage at the age of 59. Additionally, he had hypertension, dyslipidemia, and obstructive sleep apnea, which were diagnosed at the time of the surgery and had been regularly monitored at a local clinic. He had no history of prostatitis, benign prostatic hypertrophy, or sexually transmitted infections. A lumbar magnetic resonance imaging (MRI) conducted five months before admission revealed severe spinal canal stenosis at the L4/5 level with compression of the cauda equina (Figure 1). Although he was managed with symptomatic treatment, his symptoms did not improve, leading to his admission for bilateral decompression surgery at the age of 72. The surgery was completed without complications, and a urethral catheter was placed from the day of surgery until the following day. No trauma was observed during the placement, insertion, or removal of the urinary catheter.

**Figure 2:**
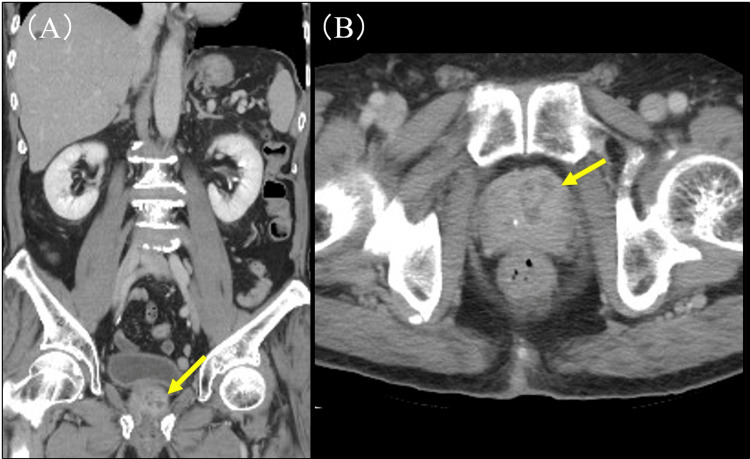
Results of contrast-enhanced CT. (A, B) Abdominal CT showing a low-density area in the prostate gland. CT: computed tomography

On postoperative day (POD) 6, he developed a fever of 38.5°C. Laboratory tests revealed elevated inflammatory markers, including leukocytosis and increased C-reactive protein levels (Table 1). Urinalysis showed pyuria (Table 2). Both two sets of blood cultures and a urine culture were positive for *M. morganii*. Contrast-enhanced computed tomography (CT) revealed a hypodense area within the prostate, leading to a diagnosis of PA (Figure 2). Levofloxacin (LVFX) 500 mg/day was initiated, to which the organism was susceptible (Table 3). Drainage with ultrasound guidance was not performed. The fever was resolved by POD 11, and inflammatory markers showed improvement by POD 17. Follow-up urine and two sets of blood cultures performed on POD 13 and 14 were both negative. Although a follow-up imaging study was not performed, LVFX was continued for a total of 21 days, during which no signs of recurrence, such as fever or pyuria, were observed. The patient was discharged on POD 27.

**Table 1:** The results of blood tests on POD 6. POD: postoperative day

Inspection items	Result	Reference
Red blood cell (RBC)	399×10⁴/μL	435-555×10⁴/μL
White blood cell (WBC)	12100/μL	3300-8600/μL
Hemoglobin	11.4 g/dL	13.7-16.8 g/dL
Platelet (PLT)	21×10⁴/μL	15.8-34.8×10⁴/μL
WBC differential %		
Neutrophils	96.10%	40-70%
Lymphocytes	2.30%	25-45%
Monocytes	1.40%	2-7%
Eosinophils	0.10%	1-6%
Basophils	0.1%	0-1%
Total protein (TP)	6.5 g/dL	6.6-8.1 g/dL
Blood urea nitrogen (BUN)	18.2 mg/dL	8-20 mg/dL
Creatinine (Cre)	0.92 mg/dL	0.65-1.07 mg/dL
Sodium (Na)	136 mmol/L	138-145 mmol/L
Potassium (K)	3.5 mmol/L	3.6-4.8 mmol/L
Chloride (Cl)	101 mmol/L	101-108 mmol/L
Estimated glomerular filtration rate (eGFR)	62.3 mL/min/1.73 m²	60 mL/min/1.73 m² or above
C-reactive protein (CRP)	3.71 mg/dL	0-0.14 mg/dL

**Table 2:** The result of urinary tests on POD 6. HPF: high-power field; POD: postoperative day

Inspection items	Result	Reference
Specific gravity	>1.030	1.005-1.030
pH	6	4.6-8.0
Protein	1+	Negative
White blood cell (urine sediment)	>100/HPF	0-5/HPF
Red blood cell (urine sediment)	5-9/HPF	0-5/HPF
Bacteria (urine sediment)	±	Negative

**Table 3:** Antibacterial susceptibility test of isolated Morganella morganii. S: susceptible; I: intermediate; R: resistance

Antibiotic	Minimum inhibitory concentration	Sensitivity
Ampicillin	>16	R
Piperacillin	<16	S
Cefazolin	>16	R
Cefotiam	>16	R
Cefmetazole	<16	S
Ceftazidime	<4	S
Cefditoren pivoxil	1	S
Cefepime	<8	S
Meropenem	<1	S
Amoxicillin-clavulanate	>16	R
Cefoperazone-sulbactam	<16	S
Piperacillin-tazobactam	<16	S
Gentamicin	<4	S
Amikacin	<16	S
Levofloxacin	<2	S
Fosfomycin	>16	R
Trimethoprim-sulfamethoxazole	<0.5	S

## Discussion

This report describes a rare case of PA related to *M. morganii*. The bacterium was identified through urine cultures and two sets of blood cultures, indicating it as the causative pathogen rather than a contaminant. Shortly before the onset of PA, the patient underwent surgery and had a urinary catheter inserted. He improved after 21 days of treatment with LVFX.

PA can manifest with a variety of local and systemic signs and symptoms [3,6]. The presentation can be nonspecific [11], and multiple symptoms are commonly reported [1]. In some cases, PA might be incidentally discovered without any symptoms [1]. PA frequently presents with symptoms related to lower urinary tract symptoms (LUTS), including incomplete bladder emptying, fever, perineal discomfort, dysuria, urinary retention, and increased urinary frequency [2,3]. PA can arise as a complication of acute bacterial prostatitis [6]. Persistent fever or a lack of improvement in maximum temperature after 36 hours of prostatitis treatment should raise suspicion for PA [3,12].

Physical examination, particularly digital rectal examination (DRE), can reveal fluctuation and tenderness; however, fluctuation is not always present, with one study reporting positive DRE findings in only 52.2% of patients [3,11].

Urinary tests, including urinalysis and urine cultures, are crucial for diagnosing PA. All men in one study demonstrated leukocytes in their midstream urine [4]. Urinalysis for leukocytes is part of the diagnostic workup [11]. More than 10 white blood cells per high-power field suggest a positive diagnosis [2]. Midstream urine is collected for culture to identify causative pathogens [4]. One study found causative pathogens in midstream urine in 11 out of 18 patients [4]. Urine culture is the preferred method for diagnosing acute bacterial prostatitis [2]. Empiric antibiotic therapy can be started immediately after collecting the urine sample, and coverage can be tailored based on culture results [4].

Other laboratory tests, such as a complete blood count (CBC), electrolyte levels, and blood culture, may be performed, especially in patients who appear systemically ill or may have impaired renal function [2]. Blood cultures were positive in 52.2% of patients in one study [3]. Inflammatory markers like white blood cells (WBC), C-reactive protein (CRP), and procalcitonin may also be assessed [3]. If surgical drainage is performed and abscess fluid is obtained, it should be analyzed for pathogens, including aerobic, anaerobic, and fungal pathogens, mycoplasma, and mycobacteria [4,6,11]. This can help identify uncommon pathogens [4].

A variety of bacterial organisms have been isolated as causative agents of PA. *Escherichia coli* (*E. coli*) is frequently identified as the predominant pathogen, noted as the most common pathogen in 60-80% of PA cases in the post-antibiotic era [1,4,12]. Other commonly reported bacterial pathogens include *Klebsiella pneumoniae*, which accounted for 11% or 17.4% of cases in different studies and is specifically mentioned as a cause of emphysematous PA [1,3,4,12,13]. *Staphylococcus aureus *is also a significant cause, historically common from hematogenous spread and remaining the most frequent cause of hematogenously spread PA [1]. Increasing cases due to methicillin-resistant *Staphylococcus aureus* (MRSA), both nosocomial and community-acquired, are a growing concern [1]. Other reported bacterial pathogens include *Enterococcus* species, with *Enterococcus faecalis *specifically noted in some cases, and *Pseudomonas aeruginosa*, identified in 5% of cases in one review [1,4,12].* Streptococcus *species are also listed, including *Streptococcus agalactiae *[1,3]. In specific populations, particularly the immunocompromised, atypical bacteria should be considered [1,4]. This group includes *Mycobacterium*, such as *Mycobacterium tuberculosis* (mTB) and *Mycobacterium avium intracellulare*; disseminated mTB is reported in human immunodeficiency virus (HIV)/immunocompromised patients and those with previous Bacillus Calmette-Guérin (BCG) therapy [1,4]. *Burkholderia pseudomallei *and *Burkholderia cepacia*, as well as *Brucella melitensis*, have also been reported as causes [1,3,6].

As far as we know, there has only been one report of *M. morganii* being isolated from PA, and it was reported as a mixed infection with *Klebsiella pneumoniae* [14]. *M. morganii *has been identified as a leading pathogen responsible for nosocomial infections in adults [9]. This bacterium is frequently found in postoperative patients and has been associated with outbreaks in healthcare settings [15]. Additionally, the use of urinary catheters may be linked to *M. morganii *infections [15]. In our case, a nosocomial infection was suspected following surgery, suggesting that PA due to *M. morganii* may have potentially developed via these infection pathways.

## Conclusions

PA can present with diverse and often nonspecific symptoms, making clinical suspicion and accurate diagnostic workup essential. The identification of *M. morganii *as the causative agent underscores the importance of considering atypical pathogens in postoperative or nosocomial settings, particularly in patients with urinary catheterization. Further studies are needed to understand the pathogenic pathways and optimal management strategies for PA caused by uncommon organisms such as *M. morganii*. Early detection and tailored treatment remain critical in improving patient outcomes and preventing complications.
